# Updates in the Management of Congenital Melanocytic Nevi

**DOI:** 10.3390/children11010062

**Published:** 2024-01-02

**Authors:** Mia A. Mologousis, Serena Yun-Chen Tsai, Kristin A. Tissera, Yakir S. Levin, Elena B. Hawryluk

**Affiliations:** 1 School of Medicine, Tufts University, Boston, MA 02111, USA; 2 Dermatology Program, Boston Children’s Hospital, Boston, MA 02115, USA; 3 Department of Dermatology, Massachusetts General Hospital, Boston, MA 02114, USA; 4 School of Medicine, Harvard University, Boston, MA 02115, USA; 5 School of Medicine, Duke University, Durham, NC 27710, USA; 6 Wellman Center for Photomedicine, Massachusetts General Hospital, Boston, MA 02114, USA

**Keywords:** congenital melanocytic nevus, melanoma, neurocutaneous melanocytosis, laser therapy, surgery, dermabrasion, cryotherapy, curettage, disease management, review

## Abstract

Congenital melanocytic nevi (CMN) carry an increased risk of melanoma and may be disfiguring, and consensus regarding treatment recommendations is lacking. While clinical monitoring is the standard of care, many caregivers are interested in its removal to prevent psychosocial burden or to decrease risk. Although melanoma can occur regardless of CMN removal, there are a variety of treatments that may offer improved cosmesis or local symptom control, including surgical excision, laser therapy, and other superficially destructive techniques. Regardless of the selected management, these patients are monitored for ongoing melanoma risk. An extensive discussion with families regarding the risks and benefits of observation versus active intervention is essential. To facilitate these discussions, we herein summarize current CMN management strategies and considerations.

## 1. Introduction

Congenital melanocytic nevi (CMN) are pigmented lesions arising from a proliferation of melanocytes in utero [1]. They present at or shortly after birth and occur in roughly 1% of all newborns [2]. Initially, lesions may be flat and tan [3]. Later, they may lighten or darken, become more raised, or develop proliferative nodules, satellites lesions, or overlying hypertrichosis. CMN are classified according to their projected adult size (PAS), where small nevi are less than 1.5 cm (cm), medium nevi are 1.5–20 cm, large nevi are 20–40 cm, and giant nevi are 40 cm or more [4]. These classifications are historically used to stratify the risk of malignant melanoma (MM) and neurocutaneous melanocytosis (NCM), with larger nevi conferring a greater risk [5,6].

There is a lack of consensus on the preferred approach to CMN management. Though we encourage clinical monitoring, many caregivers of infants with CMN are interested in removal to prevent social stigma and to reduce risk [7]. Numerous removal techniques have been proposed, but data surrounding their use remain difficult to interpret, given the diversity of presentations of CMN and the individual tolerance of the lesion and treatment risks. We therefore present an update of current CMN management strategies and considerations.

## 2. Indications for Ancillary Testing and Referral

### 2.1. Indications for Biopsy

CMN evolve with age, and change in color, thickness, and size [3,8]. However, pain, ulceration, bleeding, and growth out of proportion to the patient may be indications for biopsy [9]. Benign proliferative nodules may present at birth or over time. The presence of multiple similar papules or nodules is reassuring; a biopsy may be warranted if a particular nodule becomes excessively firm, ulcerates, or continues evolving after its initial growth period [10,11], or to manage a site that has become symptomatic.

### 2.2. Referral and Frequency of Dermatology Visits

Recommendations from a US expert group of pediatric dermatologists advocate for dermatology referral for large, giant, or multiple CMN [12]. Referrals can be delayed for single small or medium CMN without concerning findings. Larger, multiple, or changing nevi may require visits every three months for the first year of life and annually thereafter; the frequency of monitoring depends on individual factors relevant to nevus presentation, evolution, and caregiver comfort with natural evolution.

### 2.3. Screening for Neurocutaneous Melanocytosis

NCM is the rare proliferation of melanocytes within the central nervous system (CNS) in patients with CMN [5]. It occurs in 7–23% of patients with large lesions [13,14]. The prevalence associated with smaller lesions is less clear, although one single-center study demonstrated 73% of patients with three or more CMN of any size had NCM [15]. Neale and colleagues similarly found that quantity is a greater predictor of NCM than lesion size [16]. In their multi-center study, 83.3% of patients with abnormal magnetic resonance imaging (MRI) findings had four or more CMN. Having a single CMN of any size was not a strong independent predictor of disease. Common MRI findings of NCM include intraparenchymal melanosis and leptomeningeal enhancement. Children with NCM may develop neurologic symptoms like seizures or developmental delay [13], or associated diseases such as syringomyelia or brain tumors, among other non-cutaneous findings (termed CMN syndrome) [17]. They are also at increased risk of CMN-associated melanoma [5,17]. While there is a lack of consensus on specific screening recommendations, which may be influenced by regional practices and available imaging tools, NCM screening may be considered for infants with multiple CMN, any single giant CMN, or signs of neurologic change [5,12,16]. Early screening via MRI of the brain/spine is preferred to avoid the use of anesthesia and contrast (required to visualize melanin after myelination at around 6 months of age). 

## 3. Goals of Management 

CMN management ranges from clinical monitoring to extensive excision. New data regarding a relatively lower associated melanoma risk than previously reported have recently begun to shift the focus of management from melanoma prevention towards cosmetic optimization, as recommended by the CMN Surgery Network [18]. Thorough discussion with families/patients is necessary to determine individual treatment goals while weighing the risks and benefits of intervention (Table 1). 

### 3.1. Malignant Melanoma

MM risk is a major concern of caregivers and a primary consideration in CMN management. Existing data suggest MM occurs in roughly 1% of all CMN patients [6] and 2% of patients with large CMN [40]. This is less than previous estimates, likely due to the small, retrospective nature of early studies [41] and the relatively higher prevalence of the smallest, lowest risk CMN in the population. Melanoma risk is size-dependent, with giant CMN carrying the greatest risk [41]. Caregivers frequently search for ways to reduce their child’s melanoma risk. CMN removal confers a theoretical risk reduction by removing nevus cells that could undergo malignant transformation. Still, this theoretical reduction has not been confirmed by the literature, and there are reports of melanoma arising in patients with CMN who have undergone extensive treatments. 

Reasons for this are twofold: first, CNS-associated melanoma secondary to NCM remains a concern. Up to one-third of MM in children with CMN is CNS-associated [42]. Because melanocytes in the CNS can undergo malignant transformation, the removal of the cutaneous CMN does not reduce the risk of primary CNS melanoma. Second, cutaneous melanocytes can be left behind regardless of removal method [39]. Reports of recurrent nevi and MM in patients following even the surgical excision of CMN highlight this phenomenon [43,44,45,46]. Treatment-related scarring may also make developing cutaneous melanoma more difficult to detect [22]. Clinicians must counsel patients and caregivers regarding continued melanoma risk and necessary surveillance regardless of treatment.

### 3.2. Psychosocial Considerations

Psychosocial concerns are a frequent reason caregivers seek CMN treatment [47]. CMN are associated with lower self-esteem and stigmatization, particularly with larger lesions or those in cosmetically sensitive areas such as the face [20]. On one hand, up to 65% of CMN may lighten spontaneously [19], though an expectation of disappearance is not realistic. On the other, delaying treatment may complicate removal or worsen cosmesis. As lesions grow, some modalities become less effective and scar healing may be less optimal as treatment is forced to involve a larger area. Still, many patients and parents may find post-surgical scarring less socially stigmatizing [48]. In a study investigating the visual impact of large and giant congenital nevi, when the images of large to giant CMN and scarring were presented side-by-side, participants generally preferred scarring over nevi [49]. It is speculated that a less favorable response towards large nevi may be secondary to a lack of familiarity with CMN, as people tend to be more comfortable with recognizable lesions, such as scars. 

### 3.3. Other Considerations

The decision to treat CMN requires consideration of timing, balancing risks of anesthesia with the desire to prevent a child’s memory of the procedure/recovery. It is unclear precisely when children form their earliest memories, but data suggest that this may occur around the ages of 3 to 4 [50,51]. While anesthesia is used even in infants, when necessary, there are concerns about its effects on neurodevelopment [52]. Risks of voluntary sedation for the treatment of CMN must therefore be considered, particularly for treatment plans requiring multiple sedation events. 

As mentioned, size is another important consideration in choosing to treat CMN and in selecting the most appropriate technique. Logically, small- and medium-sized CMN are more amenable to treatment in general, including with excision, laser, or more superficially destructive techniques, as they involve a smaller surface area. For CMN that are small or distributed in a non-cosmetically sensitive area, surgical excision is preferred. Contrastingly, large and giant CMN may be less amenable to complete surgical excision depending on the site of involvement. Similarly, destructive techniques applied to a larger surface area to treat these large to giant CMN will result in the risk of scarring and incomplete treatment over a larger area. Disclosing these factors to families is of paramount importance.

## 4. General Skin Care Recommendations

### 4.1. Photoprotection

Skin care is a cornerstone of CMN management. Most importantly, photoprotection is emphasized to minimize any additional melanoma risk conferred on the nevus. However, no CMN treatments (including sunscreen) have been proven to definitively reduce melanoma risk [53], and the driver mutations for CMN-associated melanomas in giant CMN are distinct from those in conventional melanomas [54]. In general, standard recommendations for the use of sunscreens and photoprotective clothing are encouraged [55]. 

### 4.2. Xerosis

Xerosis and atopic dermatitis can develop within CMN, possibly secondary to reduced or ineffective sebaceous glands in affected skin [56]. Regular bathing and frequent fragrance-free emollient use with petrolatum and bland creams/ointments is recommended [57]. For persistently flared and/or pruritic skin, topical corticosteroids and calcineurin inhibitors can be prescribed, in addition to general skin care approaches used to treat eczematous dermatitis.

### 4.3. Hypertrichosis

CMN-associated hypertrichosis may increase over time [12]. Trimming or shaving are often adequate for temporary removal in younger patients. For older patients, waxing, threading, and chemical depilation are also options. Electrolysis and laser hair removal offer a more permanent solution but require multiple treatments and can be costly. Laser depilation can cause dermoscopic and histologic changes in CMN [58,59], but is unlikely to produce melanoma [60].

## 5. Surgical Excision

Surgical excision is the most widely used method for CMN removal. The recommended surgical approach depends upon CMN size, site, and functional considerations. For small and medium CMN, complete excision requiring a single procedure may be feasible based on anatomic location. Scarring can be less problematic in these small lesions since tissue trauma can be minimized using various excision techniques [61]. Contrastingly, large to giant CMN may require serial excisions, tissue expansion, flaps, and/or grafts for satisfactory closure, which may cause more visible/extensive changes and scarring. Algorithms for surgical approach in patients with large and giant nevi according to site have been proposed [23,62]. Merely considering size is not enough when designing a surgical strategy, especially for nevi located in cosmetically sensitive areas such as the face. Satisfactory surgical outcomes at these sites require meticulous preoperative design, including the careful assessment of aesthetic units, skin tension lines, and excision margins [63].

Each approach has its own challenges, especially in the management of large to giant CMN. Cheng and colleagues demonstrated that serial excision required more procedures but shorter operative times and hospital stays versus tissue expansion and skin grafting [64]. Grafting offered the least desirable cosmesis. Tissue expansion led to more complications, supporting previously published data [65]. Several retrospective studies recommend serial excision to achieve a favorable linear scar without complications of expanders or at graft sites [65,66]. Still, tissue expansion or grafting may become necessary in cases of CMN overlying or approaching joints, in which scar contracture could limit mobility [23].

Timing for surgical intervention is debated over concerns for patient autonomy and anesthesia risks [47,67]. Kim et al. found that a younger age is a predictor of emergence agitation from anesthesia [68]. However, 82% of families believed surgery-associated “trauma” for their child was light or very light in one survey [69]. Intervention in infants with greater skin elasticity may also produce better outcomes [70]. In another survey, 89% of caregivers and 99% of patients believed surgery should be pursued as early as possible, even when treatment did not impact quality of life [48]. Additionally, the younger the patient at time of first surgery, the greater the surgeon’s satisfaction with the results. As treatment is delayed and lesions grow in size, complete removal may become challenging and scar healing may be less optimal due to the greater surface area requiring treatment.

Regarding outcomes, Kinsler and colleagues found that surgical satisfaction was negatively correlated with PAS; 11–14% of those with a PAS greater than or equal to 20 cm felt surgery worsened their appearance, while roughly 90% with a PAS less than 20 cm felt surgery was worthwhile [19]. The excision of facial CMN was more worthwhile than the excision of CMN elsewhere.

Advantages of excision include the potential for a single procedure and improved cosmesis over other modalities like laser for small and medium CMN [71,72]. Disadvantages include its invasive nature, anesthesia requirement, the risk of infection, and possible scarring/disfigurement [73,74]. Functional impairment such as ptosis or scar contracture decreasing joint mobility can occur, particularly for lesions that are large, facial, or overlying joints [21,22,24]. Surgery does not prevent MM [75], and there have been several reports of MM, nevus recurrence, and the development of satellite lesions post-excision [43,44,45,46].

## 6. Laser Therapy

The use of lasers to treat CMN is controversial. While the excision of small and medium CMN can provide satisfactory results, the excision of large, giant, or facial lesions may be less acceptable [71,72]. Superficially destructive techniques like laser may be considered for these lesions as treatment goals shift from MM prevention towards improved cosmesis, in the setting of newer estimates of MM risk [25]. While the laser treatment of other pigmented lesions is well studied, evidence for its use in CMN remains limited [76]. Existing literature consists of low-quality evidence that is difficult to interpret given the variety of laser combinations and settings described, in addition to the variety in the clinical presentation of treated CMN [25]. 

The quality-switched ruby laser (QSRL) has demonstrated some efficacy when used independently to treat CMN, reducing lesion color to 0–20% of baseline color in all nine patients in one study [77]. The normal-mode ruby laser (NMRL) has also been utilized for CMN treatment, often in combination with a QSRL, to target both superficial and deep nevus cells [25]. The results of this laser combination have been mixed [78,79]. Yunayama and colleagues used a QSRL after one pass with a pulsed-dye laser, effectively reducing lesion color for all patients in an average of 7.7 sessions [80]. 

The QS alexandrite laser (QSAL) has also been used to treat CMN. It has demonstrated limited efficacy when used alone but better results are seen when it is used in combination with an ablative laser such as the carbon dioxide (CO_2_) or erbium (Er):YAG lasers [81,82,83]. 

Neodymium-doped yttrium aluminum garnet (Nd:YAG) is a pigment-specific laser. While one study describes its effective use for the treatment of general melanocytic nevi, authors do not characterize outcomes in patients with CMN specifically [84]. Another study describes eight patients with recurrent nasal CMN after Nd:YAG treatment [85]. Ultimately, all patients saw significant improvement after CO_2_ laser therapy. Authors attribute this to the CO_2_ laser’s eradication of hyperplastic tissue and advocate for its use in treating nodular nasal CMN over Nd:YAG. However, Nd:YAG may simply perform better in combination; Al-Hadithy and colleagues found that 77% of patients (40/52) treated with both Nd:YAG and CO_2_ laser saw minimal residual pigmentation following treatment [86]. 

CO_2_ and Er:YAG ablative lasers are often used for epithelial destruction before the targeting of deeper nevus cells with a pigment-specific laser like QSAL or Nd:YAG [25]. Few data have been gathered on these lasers, but both offer promising results. The CO_2_ laser has noticeably reduced CMN pigmentation in small studies [87,88,89]. Er:YAG therapy seems initially effective [90,91], but recurrence rates vary significantly [92]. One interesting approach proposed by Lim et al. involves the use of Er:YAG once immediately post-excision to treat any visible residual pigment for improved cosmesis [93]. In this study, 83% of patients (11/13) achieved a good to excellent global assessment scale (GAS) score at 16 weeks post-excision.

The use of copper vapor laser to treat CMN has recently been reported [94,95] but requires more investigation. Larger and longer-term studies are needed to assess the efficacy of all lasers in treating CMN. Existing data suggest that cosmetic outcomes of surgery alone or in combination with laser are preferable to those of laser therapy alone [71,72,96]. However, these comparative studies often include small CMN which are more easily treated with excision than the larger lesions, for which laser may have benefits.

One advantage of laser therapy is its noninvasive nature. It may not require general anesthesia or sedation in small or superficial treatments. Disadvantages of laser therapy include risks for scarring, dyspigmentation, infection, and alopecia [25]. Achieving satisfactory results is more challenging in darker phototypes; these patients are also at increased risk for dyspigmentation [26]. Laser therapy requires multiple treatments, which increases costs and re-exposes patients to anesthesia and pain. Recurrence and persistence of nevi following laser treatment is common [25,77]. Like excision, there are cases of MM in CMN treated with laser [97,98]. There have also been cases of benign lesions with histologic features of MM arising within CMN treated with laser, termed “pseudo-melanomas” [99,100]. Recent studies argue against previous theories that these atypias and MM were laser-induced [60].

Despite the controversy surrounding its use in CMN, laser therapy is subject to regional preferences and the availability of devices. While surgical treatment has been prevalent for CMN in the US, lasers have gained popularity in Asian countries. Culturally, Asians regard moles as physical features or aesthetic nuisances rather than a disease, which may motivate patients to seek cosmetic consultations rather than comprehensive dermatologic assessments. Clinics providing laser services are highly accessible in Asian countries, and the industry continues to expand at a phenomenal speed. In the US, laser treatments can be cost prohibitive, with reported expenses upwards of $1000 US dollars per visit [101], and there may be additional operating room and anesthesia charges. In Japan and Taiwan, laser therapies are more attractive than traditional surgical procedures because they are less expensive, relatively non-invasive, and have fewer complications and shorter recovery times [102].

Regarding clinical practices, physicians in Asia may be less concerned about risk of malignant transformation when using lasers on patients with small-to-medium nevi, because the incidence of melanoma is comparatively low in this ethnic group [103]. Clinical implications are that lasers are safe to treat melanocytic nevi in patients with type IV–VI skin, providing that these lesions are not located in acral areas and that the patient has no family history of melanoma [103]. Adverse effects of lasers remain a major concern in this population. Asian skin is prone to post-inflammatory dyspigmentation, which occurs sooner, lasts longer, and is present in greater magnitude in this population [104]. Excessive fluences also more easily result in dyspigmentation, scarring, and burns in patients of color [105]. As a result, clinicians in Asia prefer to perform laser procedures at lower fluences and instead plan for additional therapy sessions to avoid any unintended postprocedural complications. 

Future research on laser therapy for CMN should focus on both short- and long-term outcomes, including recurrence rate, adverse events, and the risk of MM. Furthermore, the performance of laser therapy across diverse racial or ethnic groups must be examined, considering differences in skin types and cultural factors.

## 7. Other Superficial Destructive Therapies

Many other superficial destructive techniques have been used to treat CMN. Risks common to these techniques are reviewed in Section 7.6, “Consequences of Superficial Destructive Techniques”, below.

### 7.1. Chemical Peels

Chemical peels have only treated a limited number of pigmented nevi since 1912 [22]. Ruiz-Maldonado and colleagues described the use of a phenol peel and nightly 4% hydroquinone to avoid repigmentation in 17 patients [31]. Cosmetic outcomes were not discussed, but authors asserted that acetic acid peels were less effective in depigmenting lesions than phenol peels based on prior experience. In another study, 75% of patients (15/20) treated with phenol peels reported satisfactory appearance [106]. Risks may vary in severity from superficial and temporary discomfort to reported cases of cardiac toxicity from the systemic absorption of phenol peels [32]. Chemical peels are also associated with comedone/milia development and photosensitivity [33].

### 7.2. Cryotherapy

Cryotherapy for the treatment of CMN is an area of active study. Early data lacked clear outcomes [22]. A recent study by Elmelegy reported 42 patients with CMN of various sizes treated with cryotherapy [34]. Two-thirds of patients (28/42) demonstrated excellent responses; none had poor results. The cohort reportedly had less pain than seen with other destructive therapies, which Elmelegy attributed to cryotherapy’s anesthetic effect on nerve endings. Relatedly, cryotherapy can cause local nerve damage [35]. Hypopigmentation is also frequent following cryotherapy and is more evident in individuals with darker skin tones. This can cause permanent hypopigmentation even when treating small CMN, resulting in a less desirable appearance compared to the original pigmented lesion [36].

### 7.3. Curettage

Curettage is typically performed within two weeks of life to takes advantage of a temporary cleavage plane between the superficial and deep dermis [27]. Despite limited data, existing studies demonstrate initial improvement with some repigmentation and hypertrichosis afterwards [107,108,109]. Skin grafting post-procedure to limit infection and scarring has been proposed and exhibits improved cosmesis [28].

### 7.4. Dermabrasion

Dermabrasion similarly relies upon a temporary dermal cleavage plane, making it most effective when performed early in life [30]. In the largest study of dermabrasion for CMN, Rompel et al. describe a pigmentation reduction to 0–20% of the original CMN in patients treated as newborns. The results were interestingly best for large and giant nevi; even resultant scarring was reported as satisfactory. As with curettage, concomitant grafting has been suggested to improve appearance [28,29]. However, dermabrasion has been associated with frequent repigmentation and is therefore falling out of favor [18].

### 7.5. Electrosurgery

Electrosurgery has rarely been used to treat CMN. In 1930, Stratton described the favorable cosmetic outcome of a giant nevus treated with electrocoagulation [110]. The electrodessication of 344 pigmented nevi by Walton and Cox demonstrated excellent cosmetic results [111]. Subsequent biopsies of 196 of these lesions at varying intervals revealed no malignant transformations. However, this study included only 11 pediatric patients and did not categorize findings by nevus type; CMN-specific results are therefore unclear. Of note, electrosurgery poses a risk of electric shocks/burns and the malfunction of implanted cardiac devices [37].

### 7.6. Consequences of Superficial Destructive Techniques

Superficial destructive techniques increase the risk of scarring, dyspigmentation, infection, and alopecia [33,35,37,38]. They are associated with the recurrence/persistence of nevi and involve multiple treatments [6,39]. They may require the use of general anesthesia or sedation based upon patient age, pain tolerance, and lesion size/site. 

Concerning the risk of MM following nevus biopsy, long-term monitoring is warranted. In theory, removing the epidermal portion of the lesion should partially reduce the risk of MM. However, nevus cells in the deep dermal layer still have the potential to migrate to the surface and become malignant [112], and the risk of CNS-associated melanoma secondary to NCM remains unchanged, as previously discussed. Additionally, clinical and histologic changes from these superficially destructive techniques, such as scarring, may complicate melanoma detection post-treatment [113]. 

In a study that examined the occurrence of melanoma arising from incompletely excised dysplastic nevi in 498 patients, it was found out that 6 of 304 cases (2.0%) subsequently developed melanoma at the same site [114]. Several factors could contribute to these consequences, such as sampling error from incomplete biopsies, misdiagnosis due to histologic similarities between melanoma and recurrent nevi, and the true malignant transformation of residual cells following biopsy [115]. In summary, superficial destructive therapies should only be pursued after clinicians carefully consider the risk of malignancy in each individual, taking into account factors such as skin phototype, history of sunburn, and family history of malignancy. A comprehensive discussion of risks/benefits between patients, families, and clinicians is essential in deciding to proceed with treatment.

## 8. Radiation

Information regarding use of radiation for CMN treatment is anecdotal at best. A single documented attempt in 1921 was unsuccessful, despite prior claims that radiotherapy could remove pigmented nevi [22]. Radiation therapy for skin disease poses risks of scarring, dyspigmentation, CMN recurrence/persistence, secondary malignancy, dermatitis, photosensitivity, and alopecia [116].

## 9. Future and Emerging Therapies

### 9.1. Hydrosurgery

Hydrosurgery, a high-pressure, water-based jet system that utilizes the action of water flow and the Venturi effect to enable precise excision, was proposed as an alternative to dermabrasion for the treatment of giant CMN by Coyette and colleagues in 2014 [117]. As with curettage, hydrosurgery was performed perinatally in their four patients to take advantage of the temporary dermal cleavage plane. With variable follow-up, three patients saw little residual pigment and scarring; one patient saw complete recurrence. Authors reported the technique’s ease of use and the homogeneity of results.

### 9.2. Local and Topical Therapies

Activated neuroblastoma rat sarcoma viral oncogene homolog (NRAS) mutations are found in roughly 80% of CMN [118]. Inhibitors of this pathway are therefore of interest in their treatment [119]. One translational study created mouse models of NRAS-mutant nevi mimicking CMN and found that locally injected mitogen-activated kinase (MEK), phosphoinositide 3-kinase (PI3K), and receptor tyrosine kinase (c-KIT) inhibitors and topical squaric acid dibutylester (SADBE) regressed nevi [120]. Topical SADBE also prevented melanoma in all mice. While the authors acknowledged the structural dissimilarities between mouse and human nevi, the data are encouraging for future studies of agents that target kinases downstream of RAS for therapeutic effect.

### 9.3. Gene-Targeted Systemic Therapies

Genetics have not traditionally directed CMN treatment. It is postulated that therapies targeted at their specific gene alterations may provide benefit, as NRAS and v-raf murine sarcoma viral oncogene homolog B1 (BRAF) mutations are common even in benign CMN before any progression to MM. Trametinib, a selective MEK inhibitor, is approved for the treatment of BRAF^V600E^-mutant melanoma and may also be effective in treating other melanoma subtypes, such as those with NRAS mutations [121]. Thus far, the use of trametinib in the treatment of giant CMN has only been described in a few cases. In one, a 7-year-old girl with giant CMN with an AKAP9-BRAF fusion mutation had a significant improvement in the thickness, rugosity, and nodularity of her lesion after six months on 0.5 mg of trametinib daily; however, the lesion did not lighten or shrink [122]. Similarly, trametinib significantly improved pain and pruritus in another 4-year-old girl with giant CMN and a CUX1-BRAF fusion mutation after only two weeks of therapy; fading of the lesion was noted after 22 months of treatment [123].

## 10. Observation

Observation remains a standard of care for all patients with CMN. Its main benefit is the avoidance of complications associated with treatment. Additionally, up to 65% of CMN may lighten spontaneously [19], potentially reducing cosmetic needs for treatment. The main disadvantage of observation is the potential for low self-esteem and/or bullying, particularly with larger lesions or those in cosmetically sensitive areas such as the face. 

Even if previously treated, we recommend the surveillance of residual lesions and treatment sites. While there is no standard approach to observation, we encourage monitoring for MM and NCM according to expert recommendations discussed in Section 2, “Indications for Ancillary Testing and Referral”.

## 11. Psychosocial Burden of Disease

It is vital to consider and manage the psychosocial burden of CMN, as many survey-based studies have demonstrated a decreased quality of life associated with this diagnosis. One study found that 53.9% of patients with giant CMN had significant psychosocial challenges [3]. Of the 29 children with giant CMN surveyed in another study, 30% reported social problems, while 25.9% reported behavioral and emotional problems, which were not necessarily related to the visibility of their nevi [3,124]. This is a widely debated subject, as many studies cite larger CMN and more visible locations as more psychologically burdensome [3,125]. More research is required to better define the relationship between size and site of nevi and their psychosocial impact [12].

In one survey of 192 patients with CMN of varying sizes, 8% experienced taunting secondary to their nevus. Fifty-four percent of adolescents with CMN reported a moderate to extremely large impact on their quality of life in another study [126]. Frequently associated psychological problems include anxiety, depression, aggressive behavior, and social difficulties. The cosmesis of giant CMN is most likely associated with this increased psychosocial distress [124]. 

The impact of giant CMN extends beyond patients themselves and to their families. In fact, most parents report psychosocial challenges of their own as a result of their child’s giant CMN [127]. In one report, 69% of mothers of children with giant CMN described the experience as “awful”, and many struggled with diagnosis acceptance [3,124]. Moreover, parents of children with neurologic sequelae and skin symptoms associated with their CMN are more likely to experience social and psychological distress [12].

Support groups and referrals to psychiatry are recommended to address social and behavioral concerns and to manage other psychosocial impacts of CMN [12,125,126]. Preventive psychological and cosmetic treatments early in life have also been proposed [124]. Further studies demonstrating efficacy of these support groups, psychological interventions, and cosmetic treatments in managing the psychosocial burden of disease are still needed.

The psychological burden of CMN and other chronic skin conditions is an area of active research with PeDRA’s ongoing “Big Study”.

## 12. Approach to Treatment Selection and Final Considerations

CMN can be managed in a variety of ways. The understanding of individualized patient/caregiver goals can aid in the discussion of the most appropriate approach to management for a given family. Factors such as a child’s ability to tolerate interventions and procedural risks must be weighed against concerns for psychosocial burden. Clinical monitoring can be safely recommended for all patients. Regardless of the chosen management, continued surveillance and counseling on risks of MM and NCM are essential for prevention and early detection in this population.

## Figures and Tables

**Table 1:** Methods for removal of congenital melanocytic nevi.

Method	Advantages *	Disadvantages/Risks
Extensive data exist for the following:		
None (Observation)	Up to 65% of CMN may spontaneously lighten [19], no risk of treatment complications	Psychosocial distress; for some, CMN that thicken over time, delaying treatment possibly complicating removal and impact cosmesis [20]
Surgical Excision	May require only one procedure if CMN is small to medium, improved cosmesis for small and medium CMN	Invasive; scarring/disfigurement (more significant in larger CMN and those in high-growth distribution); functional impairment from scarring/contracture formation; larger CMN may require multiple procedures for serial excisions, expanders, or grafts; infection; recurrence or appearance of new satellite lesions still possible; need for general anesthesia [19,21,22,23,24]
Limited data exist for the following: ^†^		
Laser Therapy	Noninvasive	Preferred laser combinations, settings, and frequency of treatments are not well-studied; lightens pigment rather than completely removing CMN; photosensitivity; scarring and dyspigmentation is worse in darker phototypes [25,26]
Curettage	Minimal equipment, noninvasive	Must be performed within a few weeks of life, may be supplemented with post-procedure skin grafting [27,28]
Dermabrasion	Minimal equipment, noninvasive	Must be performed within a few weeks of life; may be supplemented with post-procedure skin grafting; frequent repigmentation [18,28,29,30]
Chemical Peels	Minimal equipment, noninvasive	Cardiac toxicity from systemic absorption of phenol peels; comedone/milia development; photosensitivity; acetic peels generally less effective than phenol peels [31,32,33]
Cryotherapy	Minimal equipment, noninvasive, possible anesthetic effect causing less pain compared to other modalities [34]	Local nerve damage; hypopigmentation is common; scarring and dyspigmentation is worse in darker phototypes [35,36]
Electrosurgery	Minimal equipment, noninvasive	Electric shocks and burns; malfunction of implanted cardiac devices [34]

Abbreviations: CMN, congenital melanocytic nevi. * Treatment of congenital melanocytic nevi (CMN) theoretically reduces lifetime risk of malignant melanoma (MM) by removing/destroying cutaneous nevus cells. However, there are reports of MM even after surgical excision of CMNs. One-third of MMs in patients with CMN may also involve the central nervous system secondary to neurocutaneous melanocytosis. Scarring from removal may mask developing MM, further complicating detection. ^†^ These destructive techniques all increase the risk of scarring, dyspigmentation, infection, and alopecia [33,35,37,38]. They are associated with recurrence/persistence of nevi and involve multiple treatments in larger nevi [6,39]. All may require the use of general anesthesia or sedation based upon patient age, pain tolerance, and lesion size/site. These techniques are generally not preferred in smaller to medium CMN in which surgical excision likely offers better cosmesis.

## Data Availability

No new data were created or analyzed in this study. Data sharing is not applicable to this article.
